# Dent inclue dans la fosse nasale

**DOI:** 10.11604/pamj.2017.26.107.11221

**Published:** 2017-02-28

**Authors:** Karim Nadour, Abdelhamid Messary

**Affiliations:** 1 Service d’ORL et de CCF, Hôpital Militaire Moulay Ismail, CP 50000, Meknès, Maroc

**Keywords:** Epistaxis, ectopie dentaire, fosses nasales, Epistaxis, dental ectopy, nasal cavity

## Image en médecine

Il s'agit d'une patiente âgée de 17 ans, sans antécédents pathologiques particuliers qui a consulté pour des épistaxis à répétitions de faibles abondances par la fosse nasale gauche associées à une sensation de nez bouché homolatérale et des épisodes de rinorrhées fétides, évoluant depuis 7 mois. L'examen clinique retrouvait une patiente en assez bon état général. L'examen des fosses nasales a retrouvé quelques caillots de sang au niveau de la fosse nasale gauche, la canule d'aspiration buttait contre une formation blanchâtre de consistance osseuse implantée dans le plancher de la fosse nasale gauche et faisant saigner la muqueuse nasale en son contact. L'examen buccodentaire notamment celui de l'arcade dentaire supérieure n'a pas objectivé d'anomalie morphologique ou en nombre. Un blondeau-scanner réalisé en acquisition spiralée en coupes coronales (A) et axiales (B) en fenêtre osseuse a objectivé la présence d'un processus lésionnel spontanément hyperdense au niveau de la fosse nasale gauche en regard du cornet inférieur en rapport avec une dent incluse (flèche). La patiente a bénéficié d'une extraction de cette dent par voie endoscopique après une anesthésie locale obtenue par un bon méchage à la xylocaine naphazolinée. Les suites opératoires ont été simples. L'évolution a été marquée par la disparition des épistaxis et de l'obstruction nasale. L'éruption dentaire au niveau de la fosse nasale constitue une des formes topographiques de l'ectopie dentaire. Cette situation inhabituelle doit être évoquée devant une épistaxis et une rhinorrhée purulente fétide unilatérales.

**Figure 1:**
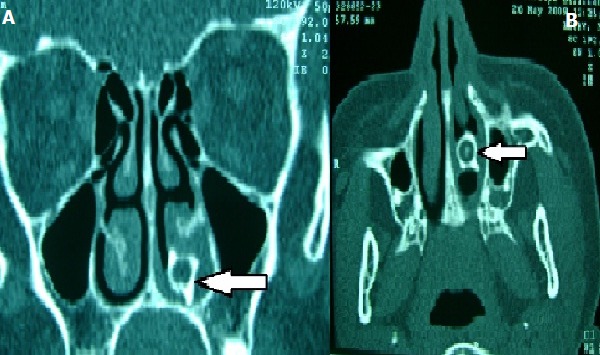
Un blondeau-scanner réalisé en acquisition spiralée en coupes coronales (A) et axiales (B) en fenêtre osseuse a objectivé la présence d’un processus lésionnel spontanément hyperdense au niveau de la fosse nasale gauche en regard du cornet inférieur en rapport avec une dent incluse (flèche)

